# Isometric artifacts from polymerase chain reaction‐massively parallel sequencing analysis of short tandem repeat loci: An emerging issue from a new technology?

**DOI:** 10.1002/elps.202100143

**Published:** 2022-05-11

**Authors:** Irena Zupanič Pajnič, Carlo Previderè, Tomaž Zupanc, Martina Zanon, Paolo Fattorini

**Affiliations:** ^1^ Institute of Forensic Medicine Faculty of Medicine University of Ljubljana Ljubljana Slovenia; ^2^ Department of Public Health Experimental and Forensic Medicine Section of Legal Medicine and Forensic Sciences University of Pavia Pavia Italy; ^3^ Department of Medicine, Surgery and Health University of Trieste Trieste Italy

**Keywords:** DNA degradation, massive parallel sequencing, PCR artifacts, STR typing

## Abstract

The recent introduction of polymerase chain reaction (PCR)‐massively parallel sequencing (MPS) technologies in forensics has changed the approach to allelic short tandem repeat (STR) typing because sequencing cloned PCR fragments enables alleles with identical molecular weights to be distinguished based on their nucleotide sequences. Therefore, because PCR fidelity mainly depends on template integrity, new technical issues could arise in the interpretation of the results obtained from the degraded samples. In this work, a set of DNA samples degraded *in vitro* was used to investigate whether PCR‐MPS could generate “isometric drop‐ins” (IDIs; i.e., molecular products having the same length as the original allele but with a different nucleotide sequence within the repeated units). The Precision ID GlobalFiler NGS STR panel kit was used to analyze 0.5 and 1 ng of mock samples in duplicate tests (for a total of 16 PCR‐MPS analyses). As expected, several well‐known PCR artifacts (such as allelic dropout, stutters above the threshold) were scored; 95 IDIs with an average occurrence of 5.9 IDIs per test (min: 1, max: 11) were scored as well. In total, IDIs represented one of the most frequent artifacts. The coverage of these IDIs reached up to 981 reads (median: 239 reads), and the ratios with the coverage of the original allele ranged from 0.069 to 7.285 (median: 0.221). In addition, approximately 5.2% of the IDIs showed coverage higher than that of the original allele. Molecular analysis of these artifacts showed that they were generated in 96.8% of cases through a single nucleotide change event, with the C > T transition being the most frequent (85.7%). Thus, in a forensic evaluation of evidence, IDIs may represent an actual issue, particularly when DNA mixtures need to be interpreted because they could mislead the operator regarding the number of contributors. Overall, the molecular features of the IDIs described in this work, as well as the performance of duplicate tests, may be useful tools for managing this new class of artifacts otherwise not detected by capillary electrophoresis technology.

**Abbreviations**
ADO: allelic dropout
AI: allelic imbalance
HDI: heterometric drop‐in
IDIs: isometric drop‐ins
LDO: locus dropout
MPS: massively parallel sequencing
ST: stutter product.

## 1 INTRODUCTION

Autosomal DNA testing is usually performed for human identification and kinship analysis, with polymerase chain reaction followed by capillary electrophoresis (PCR‐CE) of short tandem repeat (STR) markers as the gold standard [1]. In the last decade, however, new technologies, such as massively parallel sequencing (MPS), have increased the potential of forensic laboratories by enabling high‐throughput acquisition of large amounts of genetic information from a single experiment [2, 3]. In particular, MPS allows the determination of sequence variability within the STR motif and single nucleotide polymorphism (SNP) variability in their flanking regions [4, 5, 6, 7]. More recently, several kits that allow PCR‐MPS of forensically relevant STR markers have been made commercially available and validated [3]. Owing to the intrinsic properties of MPS technology, its discriminatory power has been shown to be outstanding, and this approach has therefore been proposed as an ideal tool for both mixture DNA analysis and degraded samples [8].

From a technical point of view, PCR is the first step in MPS [2, 3]. Thus, MPS may reveal the presence of well‐known PCR artifacts, such as allelic imbalance (AI), allelic dropout (ADO), stutter (ST) products, and allelic drop‐ins [9, 10, 11]. In addition, background noise sequences (i.e., molecular products showing at least one nucleotide substitution within the STR motif) are described as occurring at very low coverage, even in the analysis of high‐molecular‐weight samples [12].

A recent study performed on 75‐year‐old bone samples using the Precision ID GlobalFiler NGS STR panel kit [13] observed the stochastic occurrence of highly covered allelic drop‐ins that were named “isometric” because they had the same length as the allele that they were presumably generated from, albeit with a different nucleotide sequence. Therefore, because these drop‐ins were generated from degraded templates, they were assumed to have arisen from DNA degradation itself. However, contamination issues could not be fully excluded [13].

Thus, in this study, we aimed to test whether high levels of DNA degradation could promote the synthesis of these artifacts using damaged samples produced *in vitro*. The samples were then analyzed using the Precision ID GlobalFiler NGS STR panel kit. This study might provide valuable insights into handling a new class of artifacts otherwise not detected by capillary electrophoresis technology.

## 2 MATERIALS AND METHODS

### 2.1 DNA samples

Four DNA samples (samples A, B, FM, and TS) extracted from the blood of living men were used. Informed consent was obtained before blood collection, and the samples were anonymized. Two of the samples (TS and FM) had already been applied in other validation studies [14, 15], whereas the remaining two samples (A and B) were prepared for this study. For DNA extraction, we used the protocol described by Cigliero et al. [16], with minor modifications. Briefly, the DNA was extracted by incubation at 55°C for 4 h in 0.2 M Na–acetate (pH 7.4), 0.5% sodium dodecyl sulfate, and 100 µg/ml Proteinase K. After phenol/chloroform/isoamyl alcohol (25/24/1) purification, the samples were precipitated with ethanol (2.5 volumes), washed twice in 70% ethanol, and resuspended in double‐distilled water. A NanoDrop‐1000 spectrophotometer (Thermo Fisher Scientific, Waltham, MA, USA) was used to quantify the extracts. Replicate assessments of a 1‐µl sample were performed according to the manufacturer's user guide [17]. The final concentration of the samples was adjusted to 70 ng/µl using double‐distilled water.

### 2.2 DNA degradation and quantification

An amount of 20 µg of each sample was incubated at 70°C as described elsewhere [18]. For all but sample A, incubation was performed for 8 and 24 h (Table 1). After incubation, the samples were purified through a 3K Amicon column (Merck KGaA, Darmstadt, Germany) and resuspended in a low‐TE buffer (1‐mM Tris [pH 7.4] and 0.1‐mM Na_2_EDTA [pH 8.0]). No template degradation controls (NTDCs) were used.

**TABLE 1:** Samples employed in this study

Sample	Incubation (h)	MW	UV (ng/µl)	Auto (ng/µl)	Deg (ng/µl)	Auto/Deg	UV/Auto	PCR‐MPS
**A**	0	+++++	412	4.111 (on 1:100)	4.107 (on 1:100)	1.0	1.0	1
**A8**	8	++	205	8.401	0.012	700	24.4	2
**B**	0	+++++	586	5.951 (on 1:100)	5.061 (on 1:100)	1.2	1.0	1
**B8**	8	++	223	19.302	0.013	1,485	11.6	2
**B24**	24	+	187	0.123	<LOQ	n.c.	1,520	2
**FM**	0	+++++	582	5.731 (on 1:100)	5.619 (on 1:100)	1.0	1.0	2
**FM8**	8	++	446	26.111	0.460	56.8	17.1	2
**FM24**	24	+	322	0.049	<LOQ	n.c.	6,571	4
**TS**	0	+++++	492	4.503 (on 1:100)	4.908 (on 1:100)	0.9	1.1	1
**TS8**	8	++	554	13.702	0.019	721	40.4	2
**TS24**	24	+	443	0.033	<LOQ	n.c.	13 ,424	2

Incubation: length of the incubation at 70°C; MW: molecular weight as assessed by agarose gel electrophoresis (see Section 2 for an explanation of the scores); UV: results of NanoDrop analysis; Auto and Deg refer to the results obtained using the PowerQuant System (Promega) Auto and Deg probes, respectively; Auto/Deg: ratio between the Auto and Deg values (n.c.: not calculable); UV/Auto: ratio between the quantification data in NanoDrop analysis and the Auto probe; PCR‐MPS: number of PCR‐MPS tests performed for each sample. Untreated control samples A, B, FM, and TS were diluted 1:100 for the qPCR assay; LOQ (limit of quantification): from 50 ng/µl to 3.2 pg/µl.

Degradation was assessed by electrophoresis on 1.8% agarose gels (containing 5‐ng/ml EtBr) in the presence of molecular weight markers. Estimation of the molecular weight of the DNA samples was visually performed by considering the migration of the brightest point (BP) of the smear [14], and the following scores were arbitrarily assigned: BP > 23.1 kb: +++++; BP from 2 to 23.1 kb: ++++; BP from 1 to 2 kb: +++; BP from 0.25 to 1 kb: ++; BP < 0.25 kb: +. For DNA quantification, both NanoDrop (Thermo Fisher Scientific) analysis and quantitative PCR‐based assays were performed. The PowerQuant System kit (Promega, Madison, WI, USA) was used under the suggested conditions for each sample in duplicate [19]. Raw data were obtained using an ABI 7500 Real‐Time PCR System (Applied Biosystems, Foster City, CA, USA). The raw data were converted into Excel files using PowerQuant Analysis Software (Promega). Negative template controls and NTDCs were analyzed to verify the sterility of laboratory plastics and reagents.

### 2.3 STR typing

The Precision ID GlobalFiler™ NGS STR panel kit version 2 (Thermo Fisher Scientific) was used in this study. The DNA libraries and template preparations were run automatically on the Ion Chef System (Thermo Fisher Scientific), and an Ion S5 System (Thermo Fisher Scientific) was used for sequencing. As shown in Table S1, this method was used for duplicate analyses of 0.5‐ and 1‐ng DNA, as assessed by the Auto probe of the PowerQuant System (Promega). Seven degraded DNA samples and four untreated samples (Table S2) were amplified using 24 cycles of PCR (for a total of 16 and 5 PCR‐MPS tests, respectively). Three no‐template (NT) controls were run in the same PCR runs. Fully automated library preparation was performed using the Precision ID DL8 Kit for Chef, and barcoded libraries were pooled (50 pM) and loaded onto an Ion 530 chip according to the manufacturer's user guide [20].

Ion Torrent Suite Software 5.6 (Thermo Fisher Scientific) and Converge Software version 2.0 (Thermo Fisher Scientific) were used for MPS analysis of STR markers. The manufacturer's default relative settings were used (0.05 was applied for both the analytical and stochastic thresholds) [21], with the exceptions reported in Table S3 [22]. Default ST ratios were also applied (Table S3). The AI threshold was set at a default value of 0.35. Coverage analysis was carried out using the Coverage Analysis v 5.6.0.1 plugin. Information about mapped reads, on‐target percentage, mean depth, and uniformity of coverage were downloaded for each sample library (Barcode Summary Report file). The resulting Excel files were then used for the data analysis.

### 2.4 Data analysis and genotyping

The relative depth of coverage (rDoC) of the markers was calculated for each sample as the ratio between the mapped reads for a specific marker and the total mapped reads of the sample [13]; only the autosomal markers were considered for this analysis. To assess repeatability between duplicates, the rDoC values were compared using *r* ^2^ tests. The sequencing data for six high‐molecular‐weight DNAs, run on an Ion 520 Chip during a training test performed before this study [13], were also used as controls (therefore, our sequencing control was represented by 11 tests in total; Table S2). The average molecular weight (mw) of each of the autosomal STR markers was computed as follows: (mw of the shortest amplicon + mw of the longest amplicon)/2.

The minimum depth of coverage to assign a genotype depends on the MPS technology and the aim of the study [2]. In the current study, we set a conservative fixed value of 100× coverage as a threshold for locus call and genotype assignment. Below this cut‐off value, each specific locus was classified as “locus dropout” (LDO). This approach aims to limit the number of potentially mistyped loci [2, 3, 23, 24]. The correctness of the genetic typing was confirmed by two operators independently by comparison with the genotyping data of the corresponding untreated sample. For each sample, the occurrence of the following artifacts was scored: LDO, ADO, AI, ST, and allelic drop‐in. The frequencies of all artifacts were computed after normalization of the data (e.g., the frequencies of ADO and AI were computed based on the number of heterozygous markers having at least 100× coverage). Consistent with the aim of this study, amplicons genotyped by the software and showing a −1 or +1 repeats with respect to the original allele were scored as STs if above the ratio in Table S3.

The allelic drop‐ins were further divided into heterometric drop‐ins (HDIs) and isometric drop‐ins (IDIs). The IDIs comprised molecular products with the same length as the original allele with at least one nucleotide change within the STR motif, whereas the HDIs comprised length artifacts different from those classified as STs. The nucleotide sequences of the IDIs were compared with the published sequences of the STR alleles as catalogued in the STRSeq database [7] hosted at the NCBI BioProjects (https://www.ncbi.nlm.nih.gov/bioproject/380127; accessed: April 25, 2021). The typing of SNPs in the flanking regions was also checked.

Finally, the STR data of each duplicated test were used to build the *composite* and *consensus* profiles. *Composite* profiles were created by combining DNA profiling information from duplicate tests [25], whereas *consensus* profiles contained the genetic information confirmed in both duplicate tests [26]. To test the concordance, the resulting profiles were compared with the genotyping data of the corresponding untreated samples. After this task, the following four categories of results were identified: correct typing, incorrect typing, no typing, and profiles with more than two alleles.

### 2.5 Calculations and graphs

Microsoft Excel 2007, version 3.0.1 (Palo Alto, CA, USA) was used for calculations and graphs. The main sequencing parameters (mapped reads, on‐target percentage, mean depth, and uniformity of coverage) of the degraded samples were compared with the same parameters of the control samples using two‐tailed *t*‐tests (significance was assumed with *p* values < 0.05).

### 2.6 Comparison with IDIs found in naturally degraded samples

The main goal of the current work was to test whether *in vitro*degraded samples produced IDIs similar to the 75 IDIs first found in Second World War skeletal remains [13]. For both artificially degraded and naturally degraded samples, the following data were considered: coverage of the IDI, ratio with the coverage of the original allele, and availability of the sequence within the STRSeq database [23]. The same threshold of 100× was applied for locus calls as well.

## 3 RESULTS AND DISCUSSION

In this study, seven degraded DNA samples were produced *in vitro* (Table 1) and then tested with the Precision ID GlobalFiler NGS STR panel kit in replicated analyses (for a total of 16 tests; Tables 1 and 2). In addition, a comparison with the IDIs found in naturally degraded samples [13] was performed.

**TABLE 2:** Main features of the IDIs scored in the *in vitro* degraded samples

	Control samples	*In vitro* degraded samples	Second World War bones
**DNA samples**	10	7	16
**PCR‐MPS**	11	16	32
**DNA amount**	Average = 0.681 ± 0.226; median = 0.5; min = 0.5; max = 1	Average = 0.625 ± 0.223; median = 0.5; min = 0.5; max = 1	Average = 0.196 ± 0.170; median = 0.129; min = 0.039; max = 0.675
**Auto/Deg**	Average = 1.2 ± 0.3; median = 1.2; min = 0.9; max = 1.8; (n.c. = 0)	Average = 741 ± 584; median = 710; min = 57; max = 1485; (n.c. = 3)	Average = 29 ± 24; median = 21; min = 5; max = 82; (n.c. = 2)
**PCR cycles**	24	24	24
**Libraries (pM)**	50	50	50
**Threshold**	100×	100×	100×
**IDIs**	0	95 (1)	75 (1)
**IDIs/test (average)**	/	5.9	2.3
**Coverage**	/	Average = 272; median = 239; min = 19; max = 981	Average = 204; median = 145; min = 10; max = 1,615
**Ratio IDI versus original allele**	/	Average = 0.389; median = 0.221; min = 0.069; max = 7.285	Average = 0.350; median = 0.245; min = 0.053; max = 2.833
**Single nucleotide change**	/	92/95 (96.8 %)	64/75 (85.3 %)
**C > T**	/	84/98 (85.7 %)	72/89 (80.9 %)

For comparison, data for the IDIs found in naturally degraded samples [13] are reported in the last column together with data for the control (undegraded) samples (see Table S2 for details). DNA samples: number of DNA samples; PCR‐MPS: total number of PCR‐MPS tests; DNA amount: amount of template (in nanograms) as assessed by the Auto probe in the PowerQuant System; Auto/Deg: Auto/Deg ratio as assessed by the PowerQuant System (n.c.: number of samples for which the ratio was not calculable); PCR cycles: number of PCR cycles; Libraries (pM): concentration (in picomoles) of the pooled libraries; Threshold: threshold used for the locus call; IDIs: number of IDIs scored (in brackets, the number of IDIs corresponding to true alleles as catalogued in the STRSeq database [7]); IDIs/test (average): number of IDIs scored in each PCR‐MPS test; Coverage: coverage (in reads) of the IDIs; Ratio IDI versus original allele: ratio between the reads of the IDI and the reads of the original allele; Single nucleotide changes: number (and percentage) of single nucleotide changes scored as the source of the IDIs; C > T: number (and percentage) of C to T transitions out of the total number of nucleotide changes.

Abbreviation: IDIs, isometric drop‐ins.

### 3.1 DNA degradation and quantification

A standard hydrolytic procedure [18] was applied to the four DNA samples, allowing the production of the seven samples listed in Table 1. In agreement with our expectations, all samples exhibited severe levels of degradation, related to the length of incubation at 70°C, as assessed by agarose gel electrophoresis (Figure S1) [14] and the ultraviolet (UV)/Auto ratio [14, 18], which is the ratio between the UV‐spectrophotometric quantification and the molecular human DNA quantification as assessed using the PowerQuant Autosomal probe (84‐bp long). The Auto/Deg ratio (the ratio between the quantification values of the Auto and Deg probes of the PowerQuant kit) [19] could be calculated only for samples treated for 8 h, whereas the lack of amplification of the 249‐bp target (Deg amplicon) in all samples exposed for 24 h did not allow the calculation of the Auto/Deg ratio for these samples. NTDCs and NT samples contained no quantifiable products. Therefore, the samples were not processed further.

The degradation method employed in this study caused the hydrolysis of the phosphodiester bond of the DNA [27], enriched the molecule in apurinic–apyrimidinic sites [28], and promoted the deamination of C to U [29], which is the most common DNA lesion found in ancient DNA [30]. Although it is debatable whether our approach could mimic what spontaneously occurs on DNA in a natural environment as those based on UV exposure [31], sonication [32], and DNase I digestion [33], our approach represents a unique model for understanding the molecular mechanisms of PCR artifacts and their frequency in real casework samples.

### 3.2 Sequencing data

For the Ion 530 chip [20] used in this study, out of the addressable wells, 47.3% showed ion sphere particles (ISPs), with more than 99.1% represented by the libraries. The final library ISP percentage was 35.5, with 3.2% adapter dimers. Overall, these data are expected when sequencing degraded samples [13, 20, 23].

The PCR‐MPS of 0.5 and 1ng degraded samples could be summarized as follows. When compared with the 11 untreated test samples shown in Table S2, the degraded samples yielded, on average, fewer mapped reads (168 896 vs. 391 872, respectively; *p* value = 5.9 × 10^−6^), lower mean depth of coverage (3 610 vs. 9 612, respectively; *p* value = 3.3 × 10^−6^), lower percentage of on‐target reads (74.3% vs. 88.9%, respectively), and lower uniformity of coverage (90.5% vs. 97.6%; *p* values ≤ 0.008). However, the degraded samples showed good replicability, as indicated by the *r* ^2^ values computed from the eight duplicates (average *r* ^2^ value: 0.594 ± 0.390; median: 0.641). This result is likely due to the sufficient amount of template used for PCR amplification.

The rDoC of each of the 31 autosomal STR for degraded samples and untreated controls is shown in Figure S2. As already observed for heat‐degraded samples tested with other PCR‐MPS panels [15, 24, 34], the coverage of a few markers showed anomalously high values in the degraded samples. For example, the high‐molecular‐weight FGA marker showed an rDoC of 0.116 in the degraded samples (0.024 in the control). In agreement with Amosova et al. [35], the most likely explanation is that some sequences may be more resistant to DNA depurination on the basis of their nucleotide sequences; therefore, they are more prone to be amplified through PCR.

### 3.3 Genotyping

**FIGURE 1:**
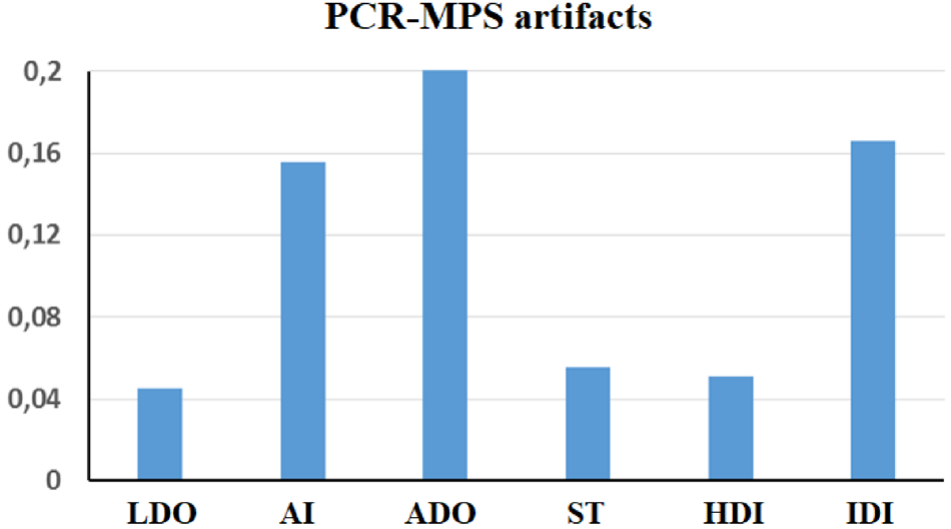
Average frequencies of the artifacts scored in the 16 PCR‐MPS tests performed on *in vitro* degraded samples (*y*‐axis). No artifacts were scored in the undegraded control samples. ADO, allelic dropout; AI, allelic imbalance; HDI, heterometric drop‐in; IDI, isometric drop‐in; LDO, locus dropout; MPS, massively parallel sequencing; PCR, polymerase chain reaction; ST, stutter product.

The genotyping data for each of the 16 degraded samples analyzed in this study are reported in Table S4, which contains the Excel files provided by Converge Software version 2.0 [21]. A comparison with the corresponding untreated sample enabled the identification of the artifacts reported in Table S1, and Figure 1 summarizes these results. On average, the frequency of LDO was approximately 4.5%, whereas AI and ADO affected approximately 15.5% and 20.0% of the heterozygous STR markers, respectively. In addition, as shown in Figure S3A (top), the occurrence of these artifacts seemed to be related to the molecular weight of the amplicons, in agreement with the model of PCR fidelity [9, 10, 11].

Among all typed markers, 30 ST products (above the threshold) were scored, along with 29 HDIs and 95 IDIs, corresponding to frequencies of 5.5%, 5.0%, and 16.6%, respectively. None of the artifacts cited earlier were scored in the untreated samples (*n* = 11) used as a control. No genotypes were obtained from the three NT controls. Thus, our current data showed that IDIs were generated from the PCR‐MPS of severely degraded samples as one of the most frequent artifacts (on average 5.9 IDIs per sample; min: 1, max: 11), and because of a high number of IDIs scored, further detailed data were acquired (see Table 2).

Regarding the molecular mechanism that generated the IDIs, a single nucleotide change was scored in 92 of 95 cases (96.8%), whereas double change events were scored in the remaining three IDIs. In total, among all nucleotide changes, 85.7% were C > T transitions, well‐known PCR artifacts [30, 36, 37, 38], mediated by the deamination of C to U [29]. C > T transitions are described as the most common errors in sequencing ancient samples [30]. As a result, these artifactual alleles usually showed more complex sequences than those of the original alleles, and as shown in Table S5, even different IDIs could arise from the same original allele in the duplicates (e.g., sample B24 at the TPOX locus, which yielded two different IDIs of allele 9). In addition, even a double IDI could arise from the original allele, as was observed for sample TS24 at locus D19S433, which yielded the original alleles 13 and 14 plus two different IDIs of allele 14. In addition, both original alleles could generate independent IDIs (e.g., sample TS8, which yielded the multi‐allelic pattern 20,20,24,24 at locus D2S1338). Interestingly, sample A8 yielded profile 12,12,15,15,15 at locus D3S4529 (original genotype: 12,15; Figure S4). Finally, only one of the 95 IDIs showed a molecular sequence corresponding to the true allelic variants cataloged in the STRSeq database [7] hosted at the NCBI BioProjects (https://www.ncbi.nlm.nih.gov/bioproject/380127; accessed: April 25, 2021). For example, allele [AATG]8 of the TPOX locus yielded three different “allele 8s” ([AATG]7 [AATA]1, [AATG]6 [AATA]1 [AATG]1, and [AATG]1 [AATA]1 [AATG]6) that were not cataloged. Even the sex‐specific markers DYS391, SRY, and Y‐InDel showed sequence artifacts.

**FIGURE 2:**
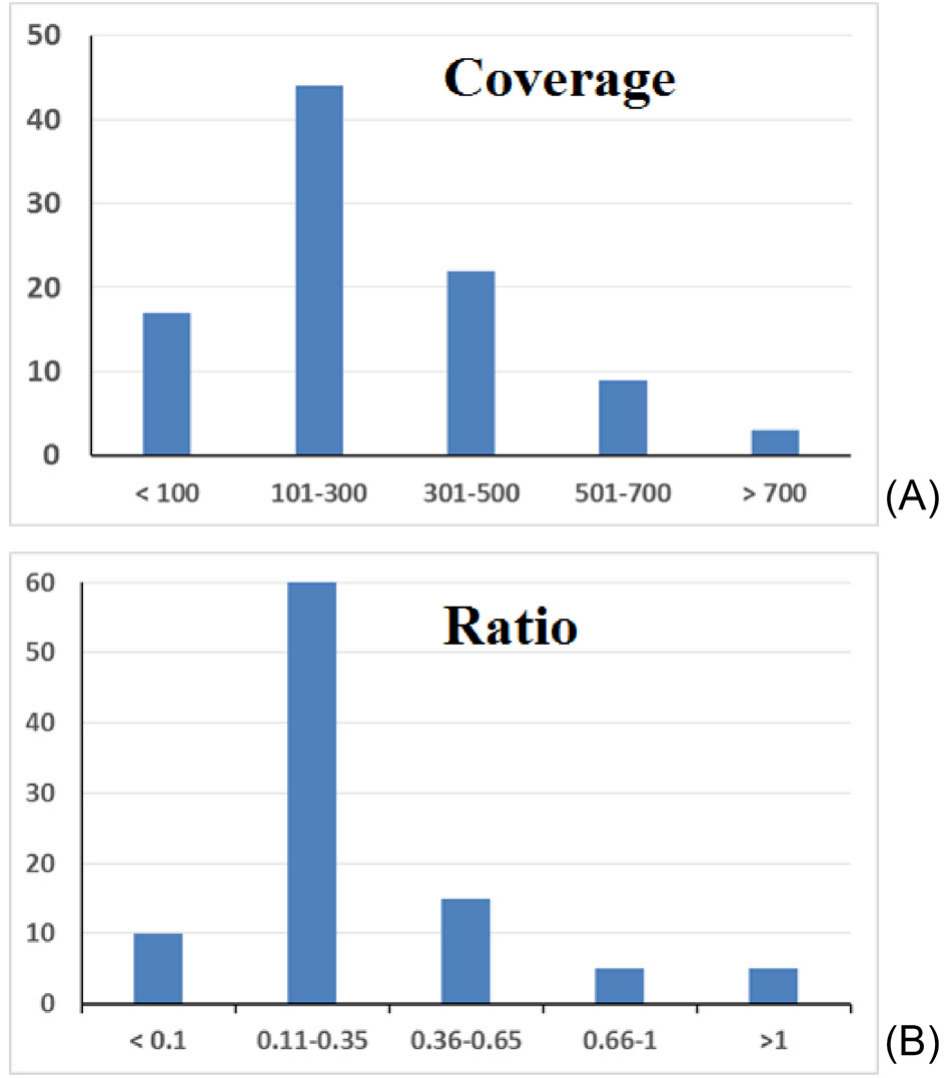
Main features of the 95 IDIs scored in this study: (A) coverage (reads) of the IDI; (B) ratio between the coverage of the IDI and the coverage of the original allele. The data were pooled into five arbitrarily set ranges (*x*‐axis) for both the coverage and the ratio; *y*‐axis: number of observations. IDIs, isometric drop‐ins.

The coverage of these artifacts ranged from 19× to 981× (average: 271 ± 190; median: 239), with 44 observations (46.3% of the total) in the range of 101× to 300× (Figure 2A, top). In addition, the ratios between the coverage of the IDI and the coverage of the original allele ranged from 0.069 to 7.285 (average: 0.289 ± 0.808; median: 0.221), indicating that in 5.2% of cases, the coverage of the spurious amplicons was even higher than that of the original amplicons (Figure 2B, bottom). As shown in Figure S3B (bottom), these artifacts originated in certain loci, such as D2S1338, D21S11, D6S474, and TPOX, suggesting that the STR motif could play a role in their synthesis. However, because six loci with the same [AGAT]n core motif sequence showed IDIs with wide frequencies ranging from 7% to 47% (Table S6), it is likely that other factors are involved as well. We speculate that these findings could depend on the level of molecular damage in the template [35] and/or the amplification conditions [9, 10], for example, primer binding sequences and annealing temperatures. Additionally, the SNPs of the flanking regions were checked for concordance. The software identified spurious reads that were mislabeled as SNPs in 17 markers in the degraded samples (Figure S4D). Taken together, these artifacts showed very low coverage, reaching no more than 10% of the reads of the original allele and could easily be identified by comparison with the corresponding untreated sample.

**FIGURE 3:**
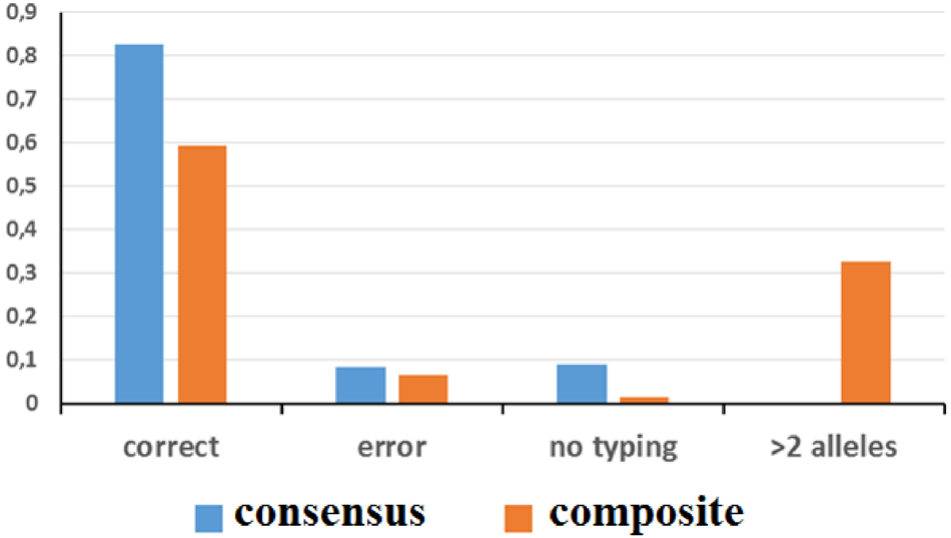
Results of STR genotyping using the *consensus* and *composite* methods. Correct: correct typing; error: incorrect typing; >2 alleles: more than two alleles per locus (see Figure S5 for the typing results for each locus); *y*‐axis: frequency. STR, short tandem repeat.

Because the data for duplicate tests were available, both *consensus* [25] and *composite* [26] profiles were generated. As shown in Figure 3, the frequency of correct typing was higher for the *consensus* profile than for the *composite* profile (82.6% vs. 59.3%), which also showed a slightly higher frequency of genotyping errors (8.3% vs. 6.6%). For the *consensus* profile, mistyping was always related to the same ADO phenomenon occurring twice, whereas for the *composite* profile, mistyping was related to ADOs (nine cases), allelic drop‐ins (seven cases), and a combination of these two phenomena (five cases). Interestingly, the *composite* profile of approximately 32% of the markers was composed of more than two alleles. As expected, these 90 multi‐allelic profiles were mainly found in loci exhibiting higher frequencies of IDIs (Figure S5). Therefore, the presence of IDIs represents a real issue, even when generating a *composite* profile from duplicate tests performed on single‐donor source samples, such as those used in this study. However, the original genotype could always be identified in each of the 90 multi‐allelic *composite* profiles.

### 3.4 Comparison with IDIs found in naturally degraded samples

Although the sample size was limited, the results presented in this paper provide an experimental explanation for the results obtained in the 75‐year‐old bone samples analyzed using the same PCR‐MPS method [13]. In particular, as shown in Table 2, the *in vitro* degraded samples were able to reproduce the principal features of the IDIs found in the naturally degraded samples, even at a higher frequency (5.9 IDIs per sample vs. 2.3 IDIs per sample). In both sets of samples, single nucleotide changes (85.3% in aged bones and 96.8% in mock samples) within the repeat arrays caused a drop in artifactual alleles. In addition, among all the nucleotide changes, the C > T transition was the most frequent in both mock samples (85.7%) and in aged bones (80.9%). However, it is likely that the main features of these artifacts (e.g., frequency, coverage) were derived from both the amount of template DNA and the DNA degradation level.

## 4 CONCLUDING REMARKS

In this study, seven samples were produced *in vitro* to test whether severe levels of DNA degradation promoted the synthesis of IDIs [13]. The PCR‐MPS results for 0.5 and 1 ng of DNA showed that IDIs were detectable only in the degraded samples, as were several other well‐characterized PCR artifacts [1, 3, 10, 11], consistent with the model of PCR fidelity [9, 39]. In addition, among the different PCR artifacts, IDIs were some of the most frequent (Figure 1), accounting for approximately 61.7% of drop‐in events.

Degraded samples are often subjected to forensic investigation by STR analysis [1, 10, 11], and PCR artifacts are known to occur in such cases. The results presented in this study supported the conclusion that a new type of drop‐in artifact, based on variations in the nucleotide sequence (IDI), could be highlighted in MPS in addition to length artifacts (HDI), which have been well characterized by PCR‐CE analysis of STR markers. The occurrence of IDIs should be considered when PCR‐MPS of STR markers is performed on aged forensic samples because these IDIs can represent actual issues, particularly if DNA mixtures need to be interpreted. The high number of IDIs that can appear in a single test (up to 11 in this study) could mislead the operator with regard to the number of contributors (Figure S4). By contrast, the artifactual origin of the IDIs should be suggested, in real casework, based on the stochastic manner in which they appear [40, 41, 42]. In addition, the unusual sequence of the IDI should also alert the operator to its spurious origin. However, this implies that duplicate tests are a reliable method for identifying these artifacts.

The molecular features of IDIs make capillary electrophoresis an unsuitable tool for identification because IDIs have the same molecular length as the original allele. By contrast, sequencing is an ideal tool for both identification and characterization. Thus, some of the potential offered by PCR‐MPS technologies could be counteracted by the occurrence of these artifactual PCR products, which are undetected by the gold standard of CE. The Ion Torrent sequencing technology employed in this study is known to be prone to insertion/deletion artifacts [43], whereas the Illumina technology is mainly subjected to misinsertions [44]. Therefore, since each platform offers its own advantages and disadvantages in STR sequencing [6, 7, 12, 13, 15, 42, 45, 46, 47, 48, 49, 50, 51], it would be beneficial to compare the outcomes of the same heavily degraded samples across different platforms. In fact, when the ForenSeq kit was used in Illumina platforms to type degraded samples [15, 46, 47, 48, 49], no IDI was scored, which could be because of the different levels of DNA degradation or the sequencing technology used. Moreover, because the data from this study suggested that IDIs were generated during the first PCR cycles, it may be interesting to investigate whether alternative kit designs containing unique molecular indices [52] can mitigate the occurrence of these artifacts.

In conclusion, although more complex assessments of larger sets of degraded samples are necessary, the results of this work provide further evidence that IDIs can be detected at measurable levels in heavily degraded samples after PCR‐MPS on the Ion Torrent platform.

## CONFLICT OF INTEREST

The authors have declared no conflict of interest.

## Supporting information

Supporting Information

Supporting Information

Supporting Information

Supporting Information

## Data Availability

The data that support the findings of this study are available in the supplementary material of this article.
