# Supplementary material for: Isometric artifacts from polymerase chain reaction‐massively parallel sequencing analysis of short tandem repeat loci: An emerging issue from a new technology?
Source: Electrophoresis. 2022 May 11;43(13-14):1521–30. doi: 10.1002/elps.202100143 (PMC9543752; doi:10.1002/elps.202100143)
Supplement: Supplementary file 1 — Supporting Information [file ELPS-43-1521-s004.pptx]

## Slide 1
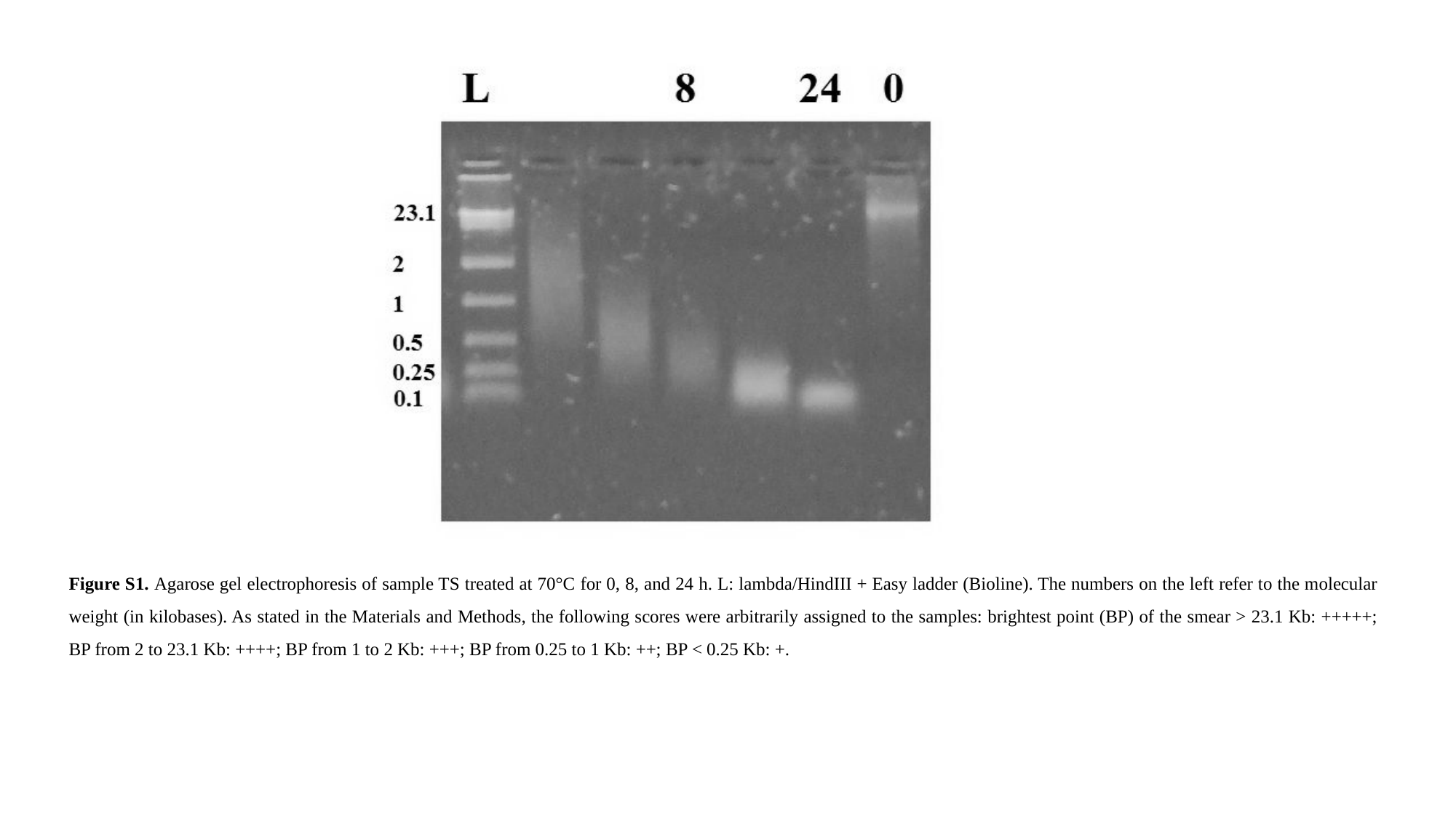

Figure S1. Agarose gel electrophoresis of sample TS treated at 70°C for 0, 8, and 24 h. L: lambda/HindIII + Easy ladder (Bioline). The numbers on the left refer to the molecular weight (in kilobases). As stated in the Materials and Methods, the following scores were arbitrarily assigned to the samples: brightest point (BP) of the smear > 23.1 Kb: +++++; BP from 2 to 23.1 Kb: ++++; BP from 1 to 2 Kb: +++; BP from 0.25 to 1 Kb: ++; BP < 0.25 Kb: +.

## Slide 2
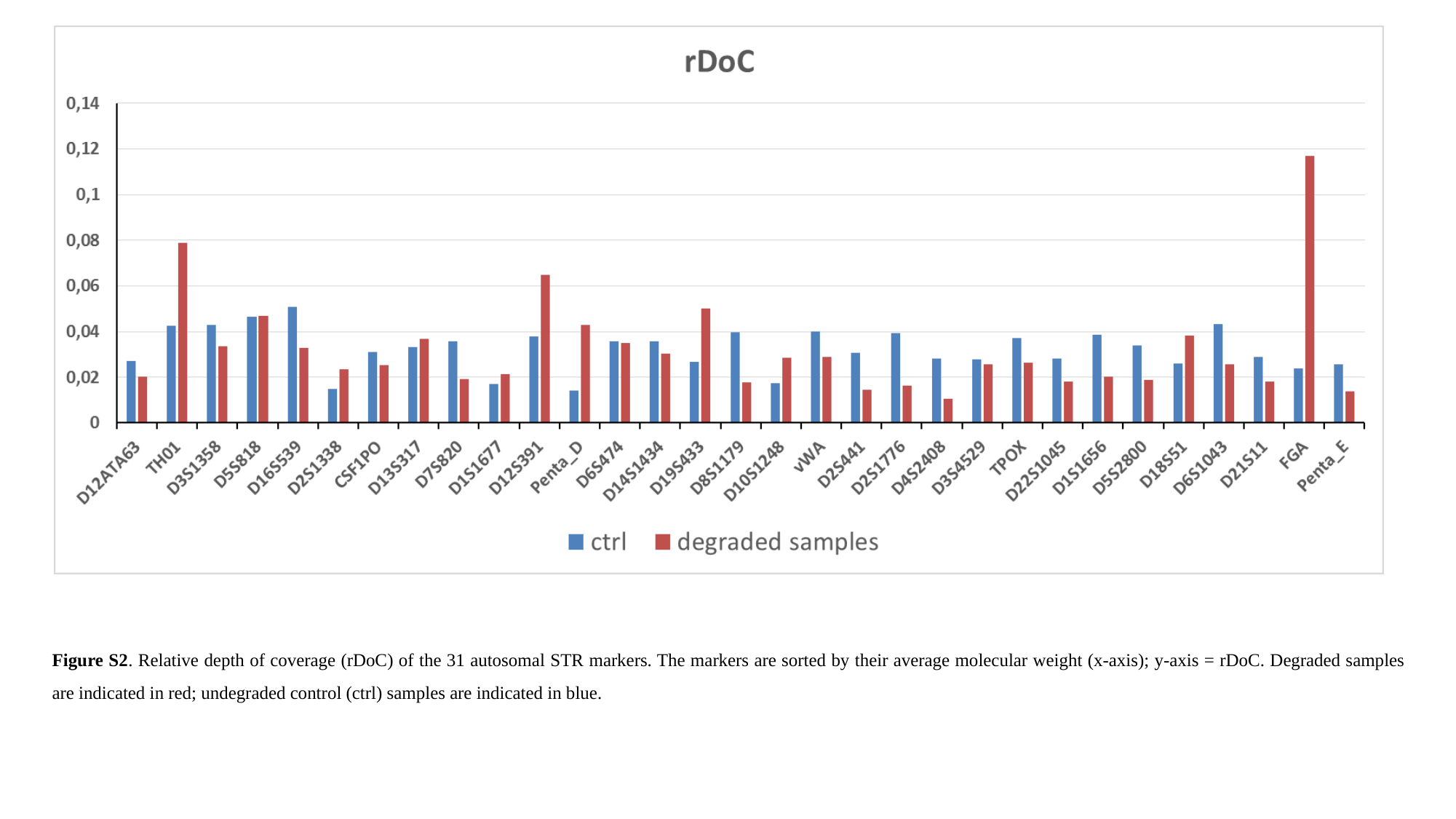

Figure S2. Relative depth of coverage (rDoC) of the 31 autosomal STR markers. The markers are sorted by their average molecular weight (x-axis); y-axis = rDoC. Degraded samples are indicated in red; undegraded control (ctrl) samples are indicated in blue.

## Slide 3
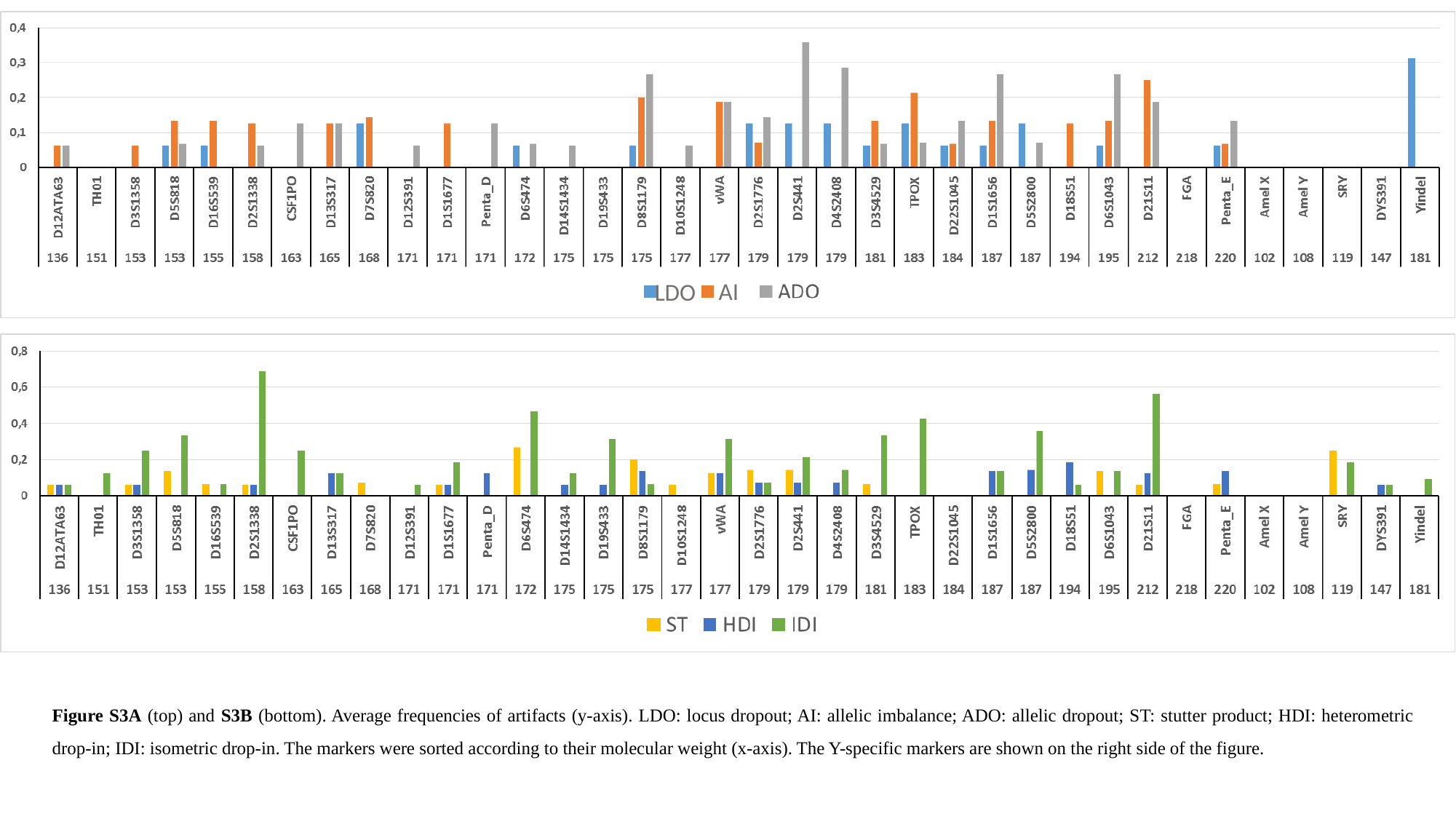

AI
LDO
Figure S3A (top) and S3B (bottom). Average frequencies of artifacts (y-axis). LDO: locus dropout; AI: allelic imbalance; ADO: allelic dropout; ST: stutter product; HDI: heterometric drop-in; IDI: isometric drop-in. The markers were sorted according to their molecular weight (x-axis). The Y-specific markers are shown on the right side of the figure.

## Slide 4
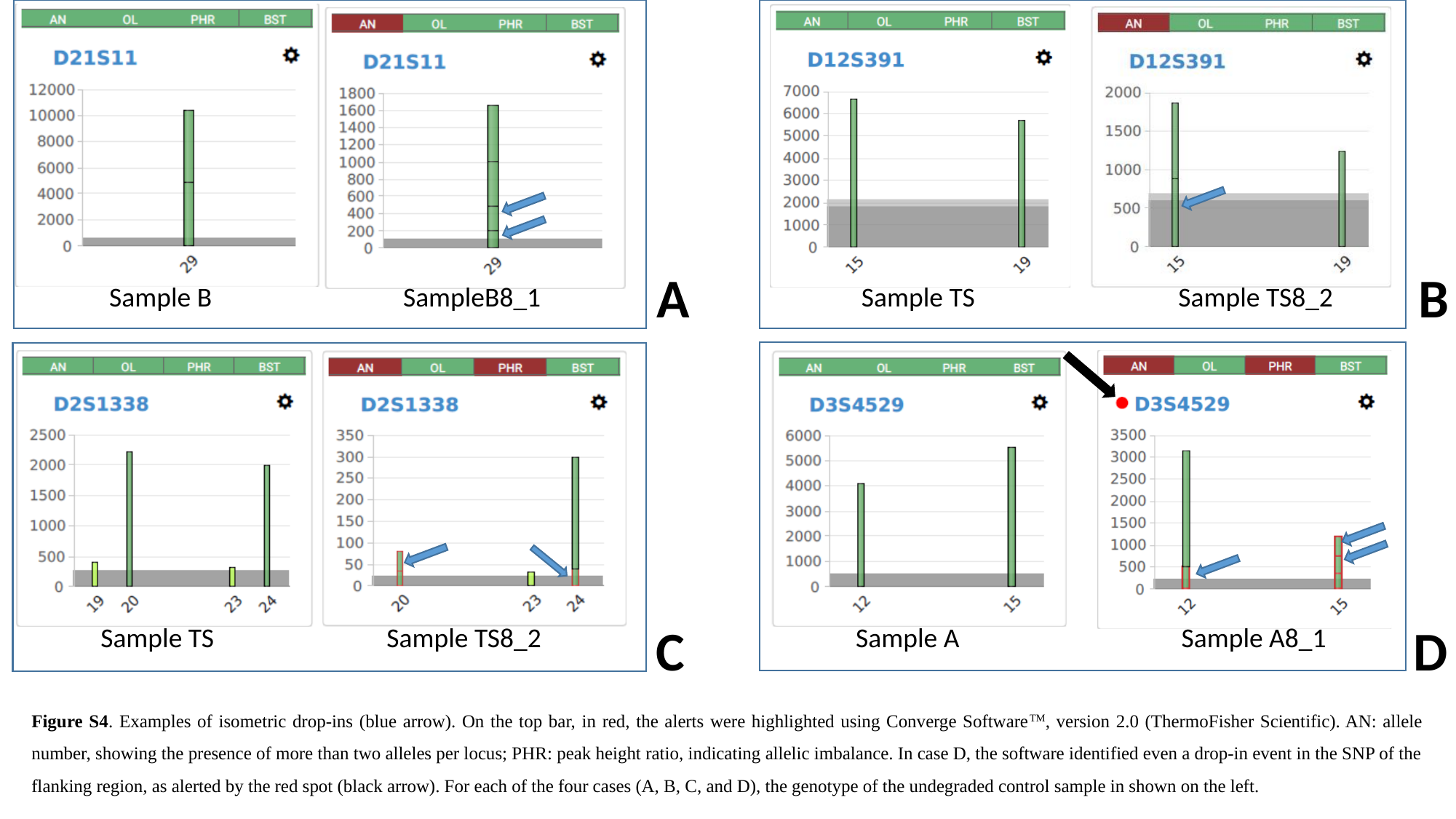

A B
Sample B SampleB8_1 Sample TS Sample TS8_2
C D
Sample TS Sample TS8_2 Sample A Sample A8_1
Figure S4. Examples of isometric drop-ins (blue arrow). On the top bar, in red, the alerts were highlighted using Converge SoftwareTM, version 2.0 (ThermoFisher Scientific). AN: allele number, showing the presence of more than two alleles per locus; PHR: peak height ratio, indicating allelic imbalance. In case D, the software identified even a drop-in event in the SNP of the flanking region, as alerted by the red spot (black arrow). For each of the four cases (A, B, C, and D), the genotype of the undegraded control sample in shown on the left.

## Slide 5
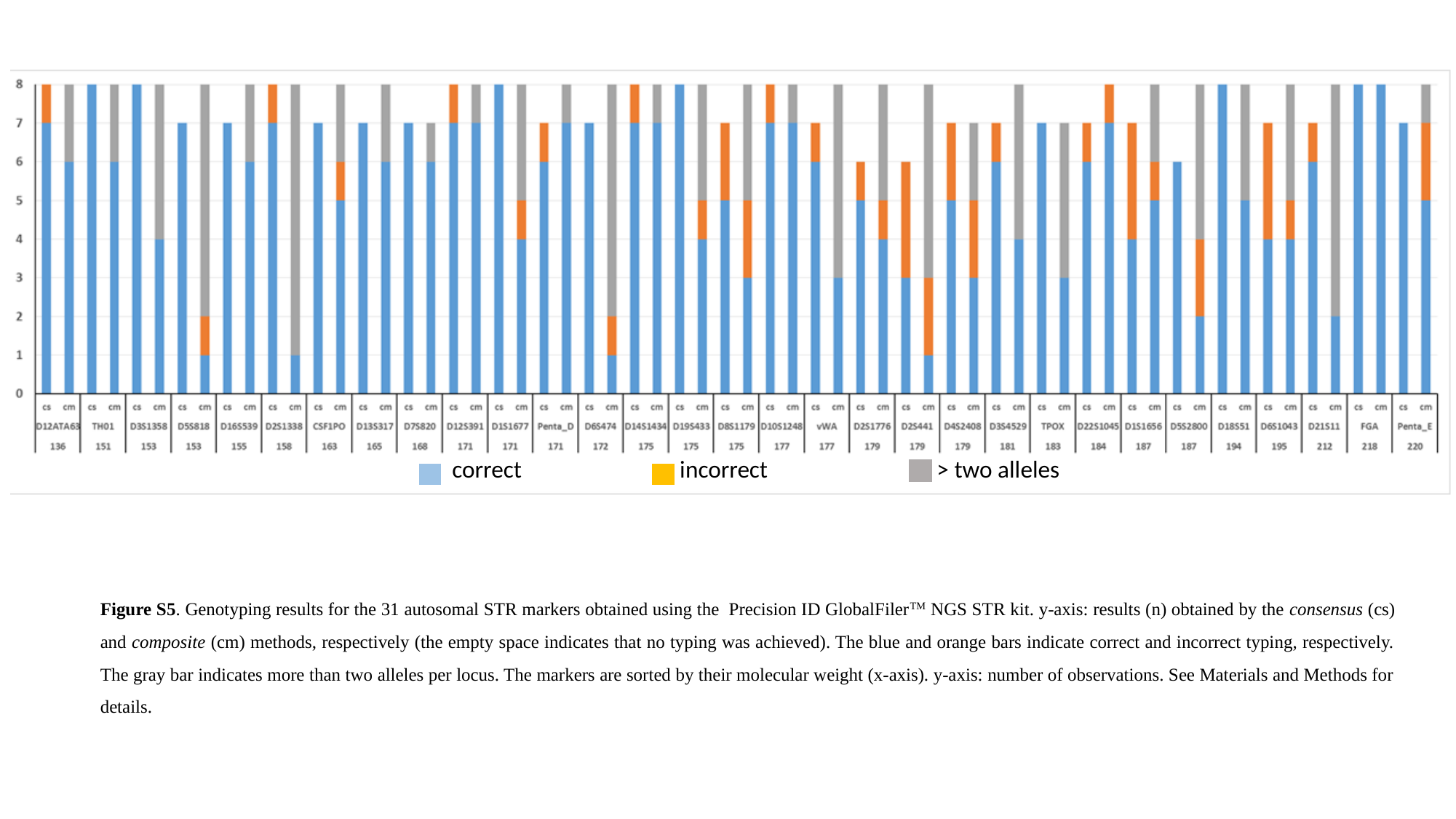

correct incorrect > two alleles
Figure S5. Genotyping results for the 31 autosomal STR markers obtained using the Precision ID GlobalFilerTM NGS STR kit. y-axis: results (n) obtained by the consensus (cs) and composite (cm) methods, respectively (the empty space indicates that no typing was achieved). The blue and orange bars indicate correct and incorrect typing, respectively. The gray bar indicates more than two alleles per locus. The markers are sorted by their molecular weight (x-axis). y-axis: number of observations. See Materials and Methods for details.
